# Trajectories of Dietary Energy, Macro and Micronutrient Intake From the Third Trimester of Pregnancy to 8.5 Months Postpartum Among Brazilian Women: The Mothers, Infants and Lactation Quality Study

**DOI:** 10.1111/mcn.70089

**Published:** 2025-08-29

**Authors:** Aline Yukari Kurihayashi, Bruna Celestino Schneider, Amanda Caroline Cunha Figueiredo, Gabriela Torres Silva, Adriana Divina de Souza Campos, Daniela Polessa Paula, Daniela de Barros Mucci, Lindsay H. Allen, Gilberto Kac

**Affiliations:** ^1^ Nutritional Epidemiology Observatory, Josué de Castro Nutrition Institute Rio de Janeiro Federal University Rio de Janeiro Brazil; ^2^ Health Science Center Serra dos Órgãos University Center Rio de Janeiro Brazil; ^3^ University of State of Rio de Janeiro, Rio de Janeiro Brazil/National School of Statistical Sciences Rio de Janeiro Brazil; ^4^ Department of Basic and Experimental Nutrition Rio de Janeiro State University Rio de Janeiro Brazil; ^5^ United States Department of Agriculture Agricultural Research Service (ARS) Western Human Nutrition Research Center Davis California USA; ^6^ Department of Nutrition University of California Davis California USA

**Keywords:** lactation, macronutrients, maternal diet, micronutrients, pregnancy, prospective study

## Abstract

Pregnancy and lactation increase maternal nutritional requirements. This study evaluated the trajectories of maternal dietary energy, macro‐ and micronutrient intake from the third trimester of pregnancy to 8.5 months postpartum, associated factors, and micronutrient intake adequacy. Longitudinal study with mother‐infant pairs recruited in a hospital in Rio de Janeiro, Brazil, during the third trimester of pregnancy. At least one 24‐h recall was answered in the third trimester of pregnancy (*n* = 369) and three visits postpartum [M1: 1.0–3.49 (*n* = 196), M2: 3.5–5.99 (*n* = 145), and M3: 6.0–8.5 months (*n* = 108)]. The dietary nutritional composition was calculated using the Brazilian Food Composition Table, and the adequacy percentage was determined based on the dietary reference intakes (estimated average requirement or adequate intake). The usual intake was determined using the Multiple Source Method, which involves fitting z‐scores with Generalised Mixed‐Effect Models. Carbohydrate and fibre dietary intake decreased 1.84 and 0.41 g, monthly, from the third trimester of pregnancy to 8.5 months postpartum. Total fat intake increased 0.89 g per month. Vitamin B2, B9, C, calcium, phosphorus and magnesium intake decreased over time, while vitamin E, selenium, and sodium increased. Prepregnancy body mass index, age, education, and income were significantly associated with changes in macro‐ and micronutrients over time. Intake adequacy was lowest at the third trimester of pregnancy for vitamin D (29.7%), B6 (53.2%) and iron (60.1%). Vitamins A and C at 8.5 months showed a significant reduction in adequacy compared to the third trimester of pregnancy. Nutritional education strategies should target pregnant women and their families during pregnancy and the postpartum period. They are essential for promoting adequate nutrition and preventing nutrient deficiencies and/or excesses that can adversely affect maternal and infant health.

## Summary

- Carbohydrate and fibre dietary intake decreased, and total fat, monounsaturated, polyunsaturated and saturated increased from the third trimester of pregnancy to 8.5 months postpartum.
- Vitamin B2, B9, C, calcium, magnesium, phosphorus and potassium intake trajectories decline over time.
- Pre‐pregnancy BMI was inversely associated with carbohydrate and fibre intake over time and positively associated with monounsaturated, saturated and total fat.
- Higher maternal age, education, and income were associated with increased micronutrient intake, except for sodium, which decreased.
- The adequacy percentage of usual intake was low for iron, vitamin D and B6 in the third trimester of pregnancy. Vitamin A and C adequacy was reduced by half over 8.5 months postpartum.

## Graphical abstract

The trajectories of energy, macro, and micronutrient intake from pregnancy to 8.5 months postpartum showed a stability in energy and protein, a decline in carbohydrates and fibre, and discrete increases in total fat, mono, poly, and saturated fat. In addition, there was a decline in the intake of most micronutrients.

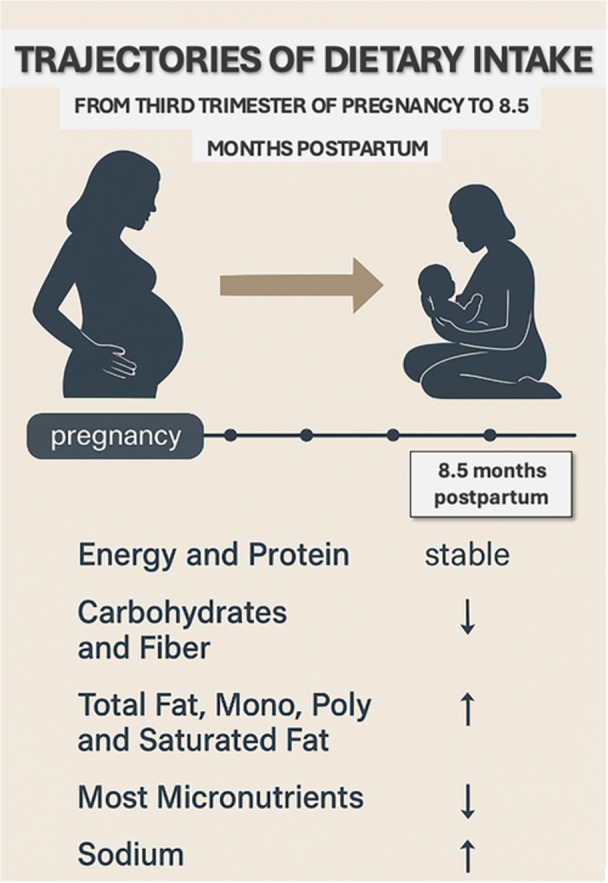

## 1 Introduction

Pregnancy and lactation are physiological periods characterised by increased energy and nutrient requirements to meet the demands of both the mother and the infant (Institute of Medicine 2005; Zeisel 2009). Indeed, adequate maternal dietary intake during these periods is critical for short‐ and long‐term maternal and infant health (Zeisel 2009; Ramakrishnan 2019; Chia et al. 2019; Taylor et al. 2023). Several studies found that an unhealthy maternal diet can be associated with gestational diabetes mellitus, pre‐eclampsia, maternal depression, low birth weight, preterm birth, and being small for gestational age (Chia et al. 2019; Opie et al. 2020; Paula et al. 2022). In addition, some studies have shown that healthy maternal dietary intake improves both the quantity and quality of human milk, which is essential for optimal infant growth and development (Allen and Hampel 2020; Petersohn et al. 2024).

Inadequate intake is frequent during pregnancy and lactation, especially in developing countries, due to relatively insufficient energy intake and infrequent consumption of nutrient‐rich foods (Lee et al. 2013). A systematic review of 17 studies revealed a significant decline in a healthier dietary pattern and an increase in discretionary food and fat intake during the transition from pregnancy to lactation (Lee et al. 2020). Furthermore, a Brazilian study involving 189 pregnant/postpartum women compared the preconception and pregnancy periods and found that the contribution to total energy intake from the unprocessed or minimally processed food group was significantly higher, and the contribution from the ultra‐processed food group was substantially lower during pregnancy compared to the preconception period (Alves‐Santos et al. 2016).

A healthy diet is adequate in energy, macro‐ and micronutrient proportions, balanced with requirements by age, sex, and physical activity levels during pregnancy and lactation, moderate in nutrient amounts, and diverse in a wide variety of foods, both between and within food groups (WHO/FAO 2024). Additionally, in Brazil, the Dietary Guidelines for the Brazilian population recommend prioritising the consumption of natural and minimally processed foods. Macronutrients are energy sources for the mother and infants, supporting tissue growth and foetal development, such as healthy brain development, and can severely impact cognitive outcomes (Mousa et al. 2019; Berger et al. 2023). In addition, micronutrients such as iron, calcium, zinc, folate, iodine, riboflavin, and B12, and vitamins A and D are essential for maternal and infant health (Institute of Medicine 2005; Allen and Hampel 2020).

Previous studies have shown that individuals with lower education levels, low income, younger age groups, and/or those who do not follow general health advice are at a higher risk of inadequate dietary intake (Doyle et al. 2017; Lee et al. 2020; Sexton‐Dhamu 2024). There is significant interest in longitudinal studies evaluating maternal diet, as it is a modifiable factor that can be altered. Additionally, over time, it is possible to identify changes, trends, and risk factors that are fundamental for promoting a healthy pregnancy and favourable perinatal outcomes. Thus, this study aims to evaluate the longitudinal trajectory of energy, macronutrients, and micronutrients in maternal dietary intake from the third trimester of pregnancy to 8.5 months postpartum, along with associated maternal factors and the adequacy of micronutrient intake.

## 2 Methods

The current manuscript presents dietary data for Brazilian women during the third trimester of pregnancy and throughout the first 8.5 months of lactation from The Mothers, Infants, and Lactation Quality (MILQ) study. The MILQ study is a prospective, multicenter cohort study conducted in Bangladesh, Brazil, Denmark, and The Gambia, designed to establish reference values for nutrient concentrations in human milk across the first 8.5 months postpartum. Detailed information about the MILQ study protocol can be found elsewhere (Allen et al. 2021).

### 2.1 Study Design and Subjects

The Brazilian arm of the MILQ study is a longitudinal study with healthy mother‐infant pairs enroled between 28 and 35 gestational weeks (baseline/antenatal visit ‐ AN) from a maternity in Rio de Janeiro during their hospital counselling visit. This timing was chosen to improve participants’ adherence to the study because it was closer to the delivery date. Participants followed four scheduled visits: within 24–72 h after birth with colostrum collection (C); 1.0–3.49 months (M1); 3.5–5.99 months (M2); and 6.0–8.5 months (M3). The MILQ protocols included data collection on anthropometry, infant and maternal body composition, maternal blood, stool, urine, breast milk volume, 24‐h dietary recalls (R24h), infant and maternal morbidity, lactation information, and Edinburgh Postnatal Depression Scales (Allen et al. 2021).

In this study, the sample consists exclusively of participants from Brazil. A total of 494 healthy women were recruited and followed up at four subsequent postpartum time points: C (*n* = 428), M1 (*n* = 235), M2 (*n* = 175), and M3 (*n* = 134). A total of 369 women responded to at least one R24h during the third trimester of pregnancy (baseline/AN), 196 at M1, 145 at M2 and 108 at M3 (Supporting Information S1: Figure S1).

The participants consisted of healthy adult women and infants. The maternal inclusion criteria comprised an age between 18 and 40 years, absence of chronic or infectious diseases, singleton pregnancy, a prepregnancy body mass index (BMI) ≥ 18.5 and < 30 kg/m^2^, a height > 145 cm, and haemoglobin levels > 100 g/L between 28 and 35 gestational weeks. Mothers should not be smokers during and after pregnancy and should not have used multivitamins and mineral supplements, except iron and folic acid, during the third trimester of pregnancy (Brazil, 2013). Moreover, women should have a low intake of fortified foods (< 1 serving per day), low regular alcohol consumption (< 30 mL per week), and avoid following a vegan or macrobiotic diet. Women who developed significant medical issues in the past or during the study (e.g., gestational diabetes or pre‐eclampsia) and discontinued exclusive breastfeeding (EBF) before M1 were excluded.

The infant's inclusion criteria comprised being born at term (≥ 37 and ≤ 42 gestational weeks), birth weight between 2500 and 4200 g, and absence of congenital malformations that interfere with feeding or growth. The exclusion criteria comprised a growth deficit, defined as having z‐scores < −2 for length‐for‐age, weight‐for‐age, or weight‐for‐length according to World Health Organization standards (WHO 2016); not being exclusively breastfed at M1; or completely stopping breastfeeding at any time during the follow‐up period.

**Figure 1:**
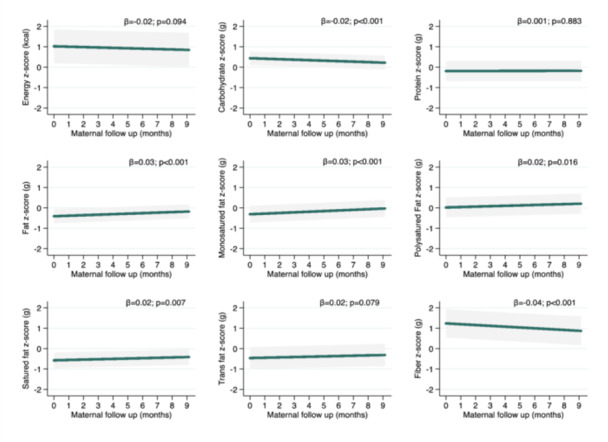
Longitudinal predictions of energy and macronutrients intake from third trimester of pregnancy to 8.5 months postpartum adjusted by usual energy (standardised), maternal age (years), maternal education (years), income (tertile), marital status (living with or living without partner), parity (primirarous or multiparous), and pre‐pregnancy BMI (kg/m^2^). *Notes:* Generalised mixed‐effect models for micronutrients intake were performed. The beta coefficient (*β*) and 95% Confidence Intervals (95% CI) are represented by the green line and grey shadow, respectively. The “0” z‐score is nutrient sample mean. Maternal follow up “0” months represents the third trimester pregnancy (baseline).

The primary reason for exclusions throughout the MILQ study was either stopping exclusive breastfeeding or stopping breastfeeding altogether (*n* = 106) (Supporting Information S1: Figure 1).

### 2.2 Dietary Assessment

Maternal dietary intake data were assessed using two R24h at each of the four follow‐up points. At baseline (between 28 and 35 gestational weeks), the first R24h was collected immediately after recruitment, followed by a second recall obtained through a phone call between 3 and 7 days later. At M1, M2, and M3, the first R24h was obtained in person during the follow‐up visit, with the second recall performed 4 days later when the participant returned to provide additional biological samples. R24h aimed to capture representative, typical days of the participant's diet. The vast majority of the R24h referred to weekdays. Therefore, R24h carried out on weekends were recorded as atypical dietary intake. If the participant reported atypical intake on the previous day, a third R24h was collected via phone. The study's longitudinal design, characterised by the continuous recruitment and follow‐up of participants throughout the year, enabled the collection of dietary data evenly distributed across all seasons.

The R24h administration was conducted using the United States Department of Agriculture (USDA) Multiple Pass Method (Conway et al. 2003), which involves five steps to minimise underreporting of food intake: 1. Quick listing of foods consumed; 2. Interviewer probes with a list of commonly forgotten foods; 3. Establishment of meal times and names given to the meals by the respondent; 4. Detailed description of foods and quantities; 5. Review of the reported intake (Steinfeldt et al. 2013). A Photographic Manual comprising 101 photos of different food sizes and portions (Crispim et al. 2018) and a Brazilian food portions reference book (Monego et al. 2013) were used to improve the accuracy of food identification and portion size estimation.

Data was recorded using the R24h Application Software (R24HR‐App), developed by the Brazilian National Survey on Child Nutrition (ENANI‐2019), to facilitate data collection and improve accuracy. The R24HR‐App includes an extensive database of food items and household measurements, covering typical and regional foods from the Brazilian population. It allows direct entry of dietary intake information on Android devices (Java‐based, compatible with Android version 5.0 or higher) and exports files in CSV format. The R24HR‐App is freely available online (https://enani.nutricao.ufrj.br/index.php/materiais/).

To ensure accuracy in the final data set, each R24h file was reviewed. Participant identifiers were cross‐checked, and fields including food descriptions, preparation types, mealtimes, units of measurement, location, and any comments were verified. All modifications were documented, and the revised files were added to the final database.

### 2.3 Energy and Nutrients

Energy, macronutrients, and micronutrients estimates were calculated using the Brazilian Food Composition Table (TBCA) (Tabela Brasileira de Composição de Alimentos TBCA 2023).

Household measures and portions were converted to mass units (g), and recipes were broken down into their ingredients. Industrialised preparations were not disaggregated into ingredients due to a lack of data on their composition, and a search was made for the food label. Nutritionally equivalent substitutions were made for food items not listed in the TBCA. Missing data (e.g., quantity or portion type) was imputed using the hot deck method (Wang et al. 2011). This method replaces missing values of a nonrespondent (recipient) with observed values from a respondent (donor) who exhibits characteristics similar to those of the nonrespondent. The method aims to ensure that the imputation preserves the structure and distribution of the original data.

Intra‐individual variability in dietary intake was adjusted using the multiple source method (MSM) (Harttig et al. 2011), which is suitable for assessing usual intake over a long period and with multiple repeated measurements. MSM applies two regression models, one for daily intake and another for event consumption, to estimate the probability of consuming on any given day and the usual amount during event consumption days. These values are multiplied to calculate the usual daily intake of each individual. In this study, the variable maternal age was added as an explanatory variation intake (an optional step). MSM is an open‐access programme available at https://nugo.dife.de/msm/.

Data quality was investigated by identifying outlier values based on the usual energy intake. R24h with energy values < 600 kcal (*n* = 22) or > 6000 kcal (*n* = 3) were removed (Oken et al. 2007). Additionally, outlier values were longitudinally assessed using the method proposed by Boone‐Heinonen et al. (2019). This method constructs a restricted cubic spline for age and employs a random‐effects model with cubic splines to extract studentized residuals. Outliers were identified based on ± 3 Standard Deviation (SD), and nine records with data from R24h were excluded. Two pregnant women and one lactating woman at M1 and one at M2 were excluded from the analysis.

The dietary markers included energy (kcal), macronutrients [carbohydrate (g and % energy contribution), protein (g and % energy contribution), total fat (g and % energy contribution), saturated fatty acids (g), monounsaturated fatty acids (g), polyunsaturated fatty acids (g), trans fatty acids (g), fibre (g)], and micronutrients [vitamin A RAE (μg), vitamin D (μg), vitamin E (mg), vitamin B1/thiamin (mg), vitamin B2/riboflavin (mg), vitamin B3/niacin (mg), vitamin B6 (mg), vitamin B12 (μg), vitamin C (mg), vitamin B9/folate equivalent (μg), calcium (mg), iron (mg), selenium (mg), magnesium (mg), phosphorus (mg), zinc (mg), copper (mg), potassium (mg), and sodium (mg)]. All dietary markers were standardised and expressed as SD.

The micronutrient intake adequacy was determined using data from Dietary Reference Intakes (DRIs) for pregnancy and lactation: Estimated Average Requirement (EAR) and Adequate Intake (AI), as defined by the Institute of Medicine (IOM) (IOM 1997; IOM 1998; Food and Nutrition Board, Institute of Medicine IOM 2000; IOM 2002; Institute of Medicine 2005; IOM 2011). The EAR represents the median value of the distribution of nutrient requirements among a cohort of healthy individuals, considering factors such as sex, age, and life stage. It is estimated to meet the needs of 50% of the population. Potassium and sodium do not have a defined EAR value (IOM 2002; National Academies of Sciences, Engineering, and Medicine; Health and Medicine Division; Food and Nutrition Board; Committee to Review the Dietary Reference Intakes for Sodium and Potassium 2019); therefore, AI values were used. The AI reference ensures nutritional adequacy, established when evidence is insufficient to develop a Recommended Dietary Allowance (RDA). The adequacy percentage (%) of usual intake was calculated for each participant and was expressed as a mean.

### 2.4 Co‐Variables

Associated factors of interest were tested using the variables described below. At baseline, the following maternal sociodemographic and anthropometric variables were collected: maternal age (18–20/21–30/31–40 years); maternal schooling (< 8, 8–12 and > 12 years); self‐reported skin colour [white, black, brown, and others (yellow/oriental and indigenous)], parity (primiparous or multiparous), family income [tertiles: first (mean = 177.9 USD dollars), second (mean = 348.8 dollars) and third (mean = 811.3 dollars)], and marital status (living with partner or living without partner).

The pre‐pregnancy body mass index (BMI) was calculated from maternal weight (kg) and height (cm) data collected from the pregnancy booklet or self‐reported at the time of recruitment. Pre‐pregnancy BMI was classified as normal weight (18.5–24.99 kg/m^2^) and overweight (25.0–29.99 kg/m^2^) (WHO 1996).

Data were collected by trained interviewers using Research Electronic Data Capture (REDCap).

### 2.5 Statistical Analyses

The first stage of the statistical analysis involved graphical checking for outlier values. R24h with energy, macro or micronutrient intake values ± 4 SD distant from the distribution were excluded. The largest number of exclusions occurred at baseline for trans‐fat (*n* = 7), vitamin B2 (*n* = 7), and vitamin E (*n* = 7). After that, sample characteristics and nutrient intake were described through the means and respective SDs.

Generalised linear mixed‐effects models (GLMMs) were fitted to assess energy and nutrient intake trajectories over time. Fractional polynomials were used separately to test departures from linearity between each outcome and time (Royston and Altman 1994). Diagnostic checks (normality of residuals and homoscedasticity) for linear mixed‐effects models were performed based on histograms and quantile‐quantile (QQ) plot analysis. Standardised intake in z‐scores (mean = 0, SD = 1) was used based on adherence tests to the model. In this approach, zero represents the sample mean, and one represents one SD above the mean. Using z‐score allows for comparative analysis of variation in nutrient intake over time, as the initial values have different ranges. Beta coefficients (*β*), 95% confidence intervals (95% CI), and *p* values were estimated using models with the Gaussian family and the “identity” link function. The equivalent *β* coefficient values (variation per month) were calculated by multiplying *β* by the nutrient's SD. Effect plots with adjusted longitudinal predictions were constructed to graphically represent the trajectories of nutrient intake from pregnancy to 8.5 months postpartum. The time was described in months, ranging from zero (baseline, during pregnancy) to 8.5 (postpartum period).

Based on the literature, the GLMM models were adjusted for energy (standardised) and a minimal but sufficient set of distal‐level confounders that affect nutrient intake (Mousa et al. 2019; Khammarnia et al. 2024). The confounders included maternal age (continuous), marital status (living with partner or living without partner), schooling (continuous), family income (tertile—originally collected continuously), parity (primiparous or multiparous—originally collected discretely), and pregestational BMI (continuous). The nature of the variables was selected according to the best fit of the statistical model. This model also identified factors associated with changes in energy, macro‐ and micronutrients over time.

Statistical differences over time for nutrient intakes and associated factors with intake trajectories were tested using the GLMM likelihood test, considering a significance level of 5% in two‐tailed tests. All analyses were conducted using Stata, version 15.1 (StataCorp, College Station, TX).

### 2.6 Ethics Statement

The Brazilian arm of the MILQ study was approved by the Research Ethics Committee of the Maternity School of the Federal University of Rio de Janeiro (1.948.992, 2.769.611, 4.449.007, approved on March 6, 2017) and the Municipal Secretary of Health and Civil Defence of the State of Rio de Janeiro (2.100.255, approved on June 5, 2017). The MILQ study (Project number: 64767717.4.3001.5279) was approved by the National Commission for Research Ethics (2.086.708, 2.875.218, 4.865.685, on May 29, 2017). The research activities were conducted following the guidelines of the Helsinki Declaration of 1975 (WMA 2013). Written informed consent was obtained from all participants.

## 3 Results

Three hundred sixty‐nine women with dietary data during the third trimester of pregnancy were studied. The mean age of the participants was 27 ± 6 years, with the majority living with a partner (81.3%) and self‐identified as brown (52.9%). Most participants had completed between 8 and 12 years of education (69.3%) and were mothers of two or more children (55.3%). Prepregnancy overweight was observed in 39% of the women (Supporting Information S1: Table S1). Participants who did not answer R24h at baseline accounted for 125 cases (25.3%), differing statistically only for maternal age groups.

The mean energy intake during the third trimester of pregnancy was (2197.0 ± 721.6 kcal) higher than 8.5‐month postpartum intake (2044.5 ± 470.2 kcal). The percentage of energy from carbohydrates decreased from 55.6% (±7.9) at the third trimester of pregnancy to 52.6% (±5.3) at M3, while the percentage of total fat increased from 31.3% (±6.3) to 33.2% (±4.6). Carbohydrate intake (g/d) decreased by 36.5 g between the third trimester of pregnancy and M3. The mean fibre intake during the third trimester of pregnancy was 26.5 g, higher than the observed intakes in M2 (23.6 g) and M3 (22.1 g). Vitamin and mineral intakes decreased from pregnancy to postpartum M3: B1 (7.7%), B2 (14.3%), B6 (11.1%), B9 (17.2%), B12 (23.1%), C (37.3%), calcium (14.7%), magnesium (12.2%), copper (18.8%), phosphorus (9.4%) and potassium (15%). In contrast, sodium intake increased by 342.9 mg (10.6%) (Table 1).

**Table 1:** Description of dietary intake during third trimester of pregnancy and 8.5 months postpartum. MILQ Study Brazil, 2024.

	Follow up period							
	Mean SD							
			Postpartum (months)					
Nutrients	Pregnancy (third trimester) (*n* = 367^a^)		M1 (1–3.4) (*n* = 195^b^)		M2 (3.5–5.9) (*n* = 144^c^)		M3 (6–8.5) (*n* = 108)	
Energy (kcal/d)	2197.0	721.6	2128.7	630.8	2088.8	638.2	2044.5^d^	470.2
Macronutrients								
Carbohydrate (%)	55.6	7.9	54.6	7.0	54.1^d^	7.4	52.6^d^	5.3
Protein (%)	16.0	3.6	16.3	3.2	16.0	3.5	16.4	2.8
Total fat (%)	31.2	6.3	31.7	5.5	32.7^d^	6.2	33.2^d^	4.6
Carbohydrate (g/d)	303.0	101.9	288.5	86.3	280.7^d^	88.4	266.5^d^	58.4
Protein (g/d)	86.7	29.2	85.2	26.6	82.2	25.9	82.8	18.3
Total fat (g/d)	76.8	31.2	75.6	28.1	76.6	29.7	76.5	24.5
Fat components								
Monounsaturated fat (g/d)	22.7	9.9	22.2	8.5	23.1	9.0	22.9	9.1
Polyusaturated fat (g/d)	18.5	9.3	18.5	8.0	18.4	7.0	18.3	6.7
Saturated fat (g/d)	25.9	9.5	25.5	10.7	24.6	10.4	25.6	7.6
Trans fat (g/d)	2.0	1.4	2.2	1.3	2.2	1.3	1.9	0.9
Fibre (g/d)	26.5	10.7	25.5	10.8	23.6^d^	9.5	22.1^d^	8.4
Vitamins								
B1 (mg/d)	1.3	0.5	1.2	0.4	1.2^d^	0.5	1.2^d^	0.5
B2 (mg/d)	1.4	0.7	1.3	0.7	1.3^d^	0.6	1.2^d^	0.5
B3 (mg/d)	18.6	10.2	18.3	8.5	17.8	6.7	18.0	7.8
B6 (mg/d)	0.9	0.5	0.8^d^	0.3	0.8^d^	0.4	0.8^d^	0.3
B9 (μg/d)	478.9	181.9	475.1	178.3	448.7	140.8	396.6^d^	127.7
B12 (μg/d)	5.2	5.3	5.4	4.6	5.5	5.0	4.0^d^	1.2
A (RAE) (μg/d)	649.5	817.2	617.5	581.7	671.7	675.8	413.4	148.4
D (μg/d)	3.5	2.7	3.5	2.8	2.8^d^	1.9	3.1	1.7
E (mg/d)	7.8	3.8	8.6^d^	4.7	8.7^d^	3.4	7.2	2.6
C (mg/d)	124.5	115.2	120.3	122.9	103.8^d^	74.8	78.1^d^	77.6
Minerals								
Calcium (mg/d)	669.0	380.5	611.7	316.0	541.3^d^	276.1	570.6^d^	261.7
Iron (mg/d)	13.7	4.9	13.6	4.5	12.7^d^	4.2	12.8	4.2
Selenium (μg/d)	44.2	19.7	46.9	21.6	44.3	20.1	44.5	15.9
Magnesium (mg/d)	294.1	96.9	280.4	89.2	267.2^d^	88.5	258.2^d^	66.9
Copper (mg/d)	1.6	1.0	1.6	0.9	1.6	0.9	1.3^d^	0.6
Zinc (mg/d)	11.2	4.5	11.3	4.0	10.7	4.2	10.7	3.4
Phosphorus (mg/d)	1264.7	423.3	1206.4	377.9	1159.5^d^	342.2	1146.4^d^	297.2
Potassium (mg/d)	2577.1	820.1	2416.4^d^	816.9	2295.1^d^	665.1	2191.7^d^	576.4
Sodium (mg/d)	3249.4	1218.2	3199.9	1092.4	3175.8	1040.1	3592.3^d^	1059.6

*Note:* Dietary Reference Intakes (DRIs), Estimated Average Requirements (EAR): Thiamin (pregnancy 1.2 mg/d, lactation 1.2 mg/d); Riboflavin (pregnancy: 1.2 mg/d, lactation 1.3 mg/d); Niacin (pregnancy 14 mg/d, lactation 13 mg/d); Vitamin B6 (pregnancy 1.6 mg/d, lactation 1.7 mg/d); Folate (pregnancy 520 μg/d, lactation 450 μg/d); Vitamin B12 (pregnancy 2.2 μg/d, lactation 2.4 μg/d); Vitamin A(RAE) (pregnancy 14–18 y: 530 μg/d | 19–50 y: 550 μg/d, lactation: 14–18 y: 885 μg/d | 19–50 y: 900 μg/d); Vitamin D (pregnancy 10 μg/d, lactation 10 μg/d); Vitamin E (pregnancy 12 mg, lactation 16 mg); Vitamin C (pregnancy 18 y: 66 mg/d | 19–50 y: 70 mg/d, lactation 18 y: 96 mg/d | 19–50 y: 100 mg/d); Selenium (pregnancy 49 μg/d, lactation 59 μg/d); Calcium (pregnancy 14–18 y: 1000 mg/d | 19–50 y: 800 mg/d, lactation 14–18 y: 1000 mg/d | 19–50 y 800 mg/d); Iron (pregnancy 14–18 y: 23 mg/d | 19–50 y: 22 mg/d, lactation 14–18 y: 7 mg/d | 19–50 y: 6.5 mg/d); Copper (pregnancy 14–18 y: 0.785 mg/d | 19–50 y: 0.8 mg/d, lactation 14–18 y: 0.985 mg/d | 19–50 y: 1.0 mg/d); Zinc (pregnancy 14–18 y: 10.5 mg/d | 19–50 y: 9.5 mg/d, lactation 14–18 y: 10.9 mg/d | 19–50 y 10.4 mg/d). DRIs, Recommended Dietary Allowances (AIs): Potassium (pregnancy 14–18 y: 2600 mg/d | 19–50 y: 2900 mg/d, lactation 14–18 y: 2500 mg/d | 19–50 y: 2800 mg/d); Sodium (pregnancy: 1500 mg, lactation: 1500 mg).

a Two exclusions during data cleaning.

b One exclusions during data cleaning.

c One exclusions during data cleaning.

^d^ Mixed‐effects Generalised mixed‐effect models likelihood *p* < 0.05. Reference category: pregnancy.

There was no change in energy and protein intake over time; however, carbohydrate intake decreased by an average of 1.84 g (*β* = −0.02 z score) per month from the third trimester of pregnancy to 8.5 months postpartum. Similarly, fibre intake decreased by 0.41 g (*β* = −0.04 *z* score) during this period. On the other hand, total fat intake increased by 0.89 g (*β* = 0.03 *z* score) each month over time, along with its fractions: monounsaturated (0.28 g), polyunsaturated (0.17 g), and saturated fats (0.21 g) (Figure 1).

**Figure 2:**
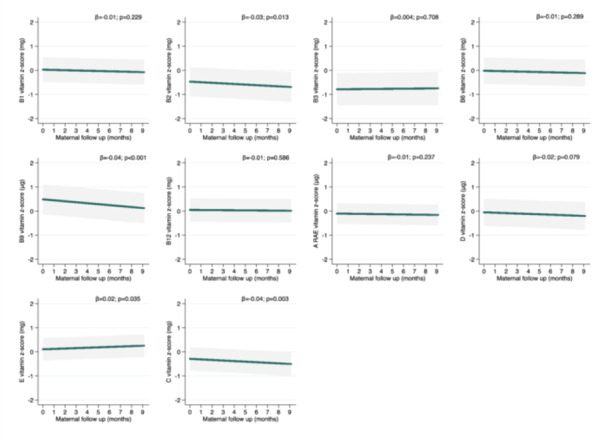
Longitudinal predictions of vitamins intake from third trimester of pregnancy to 8.5 months postpartum adjusted by usual energy (standardised), maternal age (years), maternal education (years), income (tertile), marital status (living with or living without partner), parity (primirarous or multiparous), and pre‐pregnancy BMI (kg/m^2^). Generalised mixed‐effect models for micronutrients intake were performed. The beta coefficient (*β*) and 95% Confidence Intervals (95% CI) are represented by the green line and grey shadow, respectively. The “0” *z*‐score is nutrient sample mean. Maternal follow up “0” months represents the 3rd trimester pregnancy (baseline).

The mean intake of vitamins B2, B9 and C decreased over the follow‐up period, on average, by 0.02 mg, 6.80 mcg, and 4.31 mg per month, respectively, from the third trimester of pregnancy to 8.5 months postpartum. In contrast, vitamin E increased during follow‐up by 0.08 mg (*β* = 0.02 *z* score) (Figure 2).

**Figure 3:**
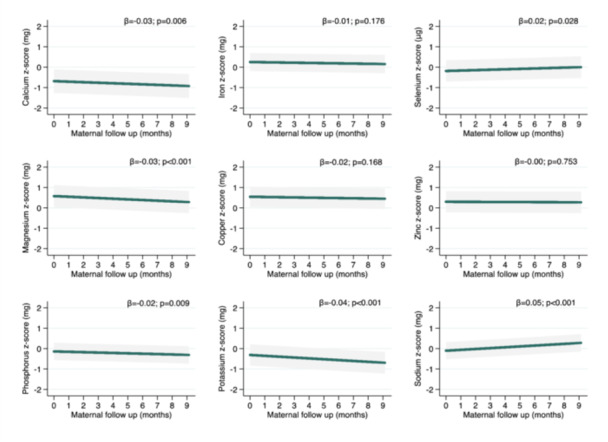
Longitudinal predictions of minerals intake according from third trimester of pregnancy to 8.5 months postpartum adjusted by usual energy (standardized), maternal age (years), maternal education (years), income (tertile), marital status (living with or living without partner), parity (primirarous or multiparous), and prepregnancy BMI (kg/m^2^). Generalised mixed‐effect models for micronutrients intake were performed. The beta coefficient (*β*) and 95% Confidence Intervals (95% CI) are represented by the green line and grey shadow, respectively. The “0” *z*‐score the nutrient sample mean. Maternal follow up “0” months represents the third trimester pregnancy (baseline).

It was observed that most minerals exhibited a reduction in mean intake over time, with the largest decreases occurring for potassium (31.1 mg; *β* = −0.04 *z* score), followed by magnesium (2.73 mg/month), calcium (10.1 mg/month), and phosphorus (7.73 mg/month). Sodium and selenium were the minerals with increased intake from the third trimester of pregnancy to 8.5 months postpartum, averaging 57.2 and 0.40 mg each month, respectively (Figure 3).

Several associations were observed between prepregnancy BMI and dietary markers. There were reductions for carbohydrates, fibre, and selenium between third trimester of pregnancy and 8.5 months postpartum. Conversely, there were increases for total, monounsaturated, and saturated fat over time (Supporting Information S1: Table S2). Additionally, higher family incomes were associated with increased vitamin E and C intake at the third income level compared to the first. Increasing maternal age increased vitamin B2, vitamin C, calcium and selenium dietary intake. Calcium intake increased, while sodium intake decreased for each year of maternal schooling. Among women living without a partner, total fat and sodium intake increased each month from the third trimester of pregnancy to 8.5 months postpartum compared to those living with a partner (Supporting Information S1: Table S2).

In the third trimester of pregnancy, the lowest adequacy percentages were observed for vitamins D (29.7%), B6 (53.1%) and E (61.3%). In the postpartum period, vitamin A showed a significant decrease in adequacy at all three evaluation points compared to pregnancy, reaching 46% of the EAR by the end of follow‐up. Similarly, vitamin C adequacy decreased from 147.1% in the third trimester of pregnancy to 74.3% at 8.5 months postpartum, while vitamin D remained far below the EAR at all postpartum points. Iron and calcium had the lowest mean adequacy percentages at the third trimester of pregnancy (60.1% and 77%, respectively). During the postpartum period, calcium adequacy remained low with a significant reduction in M2 (11 percent points), while iron significantly increased, reaching 202.9% of the EAR at M1 (Table 2).

**Table 2:** Description percentage of adequacy of micronutrients intake (%) from third trimester of pregnancy to 8.5 months postpartum. MILQ Study Brazil, 2024.

	Follow up period							
	Mean (%) SD							
			Postpartum (months)					
Micronutrients	Pregnancy (third trimester) (*n* = 367^a^)		M1 (1−3.4) (*n* = 195^b^)		M2 (3.5−5.9) (*n* = 144^c^)		M3 (6−8.5) (*n* = 108)	
Vitamins								
B1 (mg/d)	102.6	39.1	100.9	27.2	92.4^d^	33.0	95.1^d^	35.4
B2 (mg/d)	116.9	55.8	102.6^d^	47.7	97.6^d^	49.4	92.5^d^	37.7
B3 (mg/d)	122.3	56.2	136.6^d^	58.3	135.1^d^	48.0	134.2^d^	52.5
B6 (mg/d)	53.2	25.0	45.9^d^	19.1	46.0^d^	20.3	47.5^d^	18.6
B9 (μg/d)	89.5	31.1	102.9^d^	35.2	99.7^d^	31.3	88.1	28.4
B12 (μg/d)	176.4	94.3	175.4	78.0	171.5	79.8	165.1	49.1
A (RAE) (μg/d)	87.8	54.9	57.2^d^	29.1	52.8^d^	31.5	46.0^d^	16.5
D (μg/d)	29.7	18.4	29.3	17.3	26.0^d^	15.1	30.8	16.7
E (mg/d)	61.3	24.9	48.8^d^	20.3	51.8^d^	15.4	45.0^d^	16.2
C (mg/d)	158.4	123.8	103.6^d^	79.3	104.1^d^	75.3	74.2^d^	64.7
Minerals								
Calcium (mg/d)	77.1	38.4	73.9	35.3	65.9^d^	32.3	70.7	33.0
Iron (mg/d)	60.1	19.3	203.1^d^	58.6	192.8^d^	59.0	193.5^d^	58.5
Selenium (μg/d)	87.3	35.5	74.8^d^	28.7	72.1^d^	29.7	73.7^d^	23.9
Magnesium (mg/d)	96.5	28.0	105.0^d^	29.3	101.2	31.3	99.4	25.8
Copper (mg/d)	179.4	72.2	150.8^d^	55.9	153.7^d^	64.3	126.8^d^	48.1
Zinc (mg/d)	116.7	45.4	107.1^d^	35.5	100.5^d^	34.9	102.7^d^	33.0
Phosphorus (mg/d)	208.3	65.8	200.2	59.2	193.8^d^	61.9	192.9^d^	54.2
Potassium (mg/d)	86.7	25.0	84.9	26.3	82.0	22.9	78.8^d^	20.8
Sodium (mg/d)	209.4	69.8	206.7	60.0	207.7	60.4	231.6^d^	61.7

*Note:* Dietary Reference Intakes (DRIs), Estimated Average Requirements (EAR): Thiamin (pregnancy 1.2 mg/d, lactation 1.2 mg/d); Riboflavin (pregnancy: 1.2 mg/d, lactation 1.3 mg/d); Niacin (pregnancy 14 mg/d, lactation 13 mg/d); Vitamin B6 (pregnancy 1.6 mg/d, lactation 1.7 mg/d); Folate (pregnancy 520 μg/d, lactation 450 μg/d); Vitamin B12 (pregnancy 2.2 μg/d, lactation 2.4 μg/d); Vitamin A(RAE) (pregnancy 14–18 y: 530 μg/d | 19–50 y: 550 μg/d, lactation: 14–18 y: 885 μg/d | 19–50 y: 900 μg/d); Vitamin D (pregnancy 10 μg/d, lactation 10 μg/d); Vitamin E (pregnancy 12 mg, lactation 16 mg); Vitamin C (pregnancy 18 y: 66 mg/d | 19–50 y: 70 mg/d, lactation 18 y: 96 mg/d | 19–50 y: 100 mg/d; Selenium (pregnancy 49 μg/d, lactation 59 μg/d); Calcium (pregnancy 14–18 y: 1000 mg/d | 19–50 y: 800 mg/d, lactation 14–18 y: 1000 mg/d | 19–50 y 800 mg/d); Iron (pregnancy 14–18 y: 23 mg/d | 19–50 y: 22 mg/d, lactation 14–18 y: 7 mg/d | 19‐50 y: 6.5 mg/d); Copper (pregnancy 14–18 y: 0.785 mg/d | 19–50 y: 0.8 mg/d, lactation 14–18 y: 0.985 mg/d | 19–50 y: 1.0 mg/d); Zinc (pregnancy 14–18 y: 10.5 mg/d | 19–50 y: 9.5 mg/d, lactation 14–18 y: 10.9 mg/d | 19–50 y 10.4 mg/d). DRIs, Recommended Dietary Allowances (AIs): Potassium (pregnancy 14–18 y: 2,600 mg/d | 19–50 y: 2,900 mg/d, lactation 14–18 y: 2,500 mg/d | 19–50 y: 2800 mg/d); Sodium (pregnancy: 1500 mg, lactation: 1500 mg).

a Two participants excluded during datacleaning.

b One participants excluded during datacleaning.

c One participants excluded during datacleaning.

^d^ Generalised mixed‐effect models likelihood *p* < 0.05. Reference category: pregnancy.

## 4 Discussion

This study observed a steady trajectory for energy and protein dietary intake, a reduction in carbohydrate and fibre intake, and an increase in total fat intake and its fractions from the third trimester of pregnancy to 8.5 months postpartum. In general, there was a decline in micronutrient intake over time (vitamin B2, B9, C, calcium, magnesium, phosphorus and potassium), although intakes of vitamin E and selenium increased. Sodium intake increased considerably between the third trimester of pregnancy and the end of the study period. A higher prepregnancy BMI was associated with reduced fibre and carbohydrate intake and increased total, mono‐ and saturated fat intake over time. Additionally, higher maternal age (vitamin B2, C, calcium, selenium), education (calcium), and family income (vitamin C and E) were positively associated with the micronutrient trajectory, except for sodium, whose intake reduced among more educated women. Furthermore, women who lived without a partner had an increase in total fat and sodium intake over time compared to those who lived without a partner. In the third trimester of pregnancy, attention was drawn to the adequacy of iron, vitamin D, E, B6 and calcium intake, which fell below 80% of the EAR for pregnancy. At 8.5 months postpartum, the adequacy of intake for vitamins A and C had decreased by half of the EAR for lactation compared to the third trimester of pregnancy.

Pregnancy and lactation are critical biological periods that require attention to ensure an adequate supply of energy, macro, and micronutrients. A nutritionally adequate diet during these stages plays a fundamental role in child growth, cognitive development, immune system function, and metabolic programming, with an impact that lasts throughout the individual's life (WHO 2016; Victora et al. 2016; Hawkes et al. 2019; Ramakrishnan 2019; FAO/WHO 2024). This study revealed that, during the third trimester of pregnancy, the average intake of energy, carbohydrate, and fat was similar to the mean found in a meta‐analysis of 54 studies mainly carried out in America with a total of 135,566 pregnant women aged between 23 and 37 years: 2197.0 versus 2115.6 kcal, 86.7 versus 78.21 g, and 76.8 versus 74.17 g, respectively (Khammarnia et al. 2024). The protein intake in the present study was slightly lower than in other studies: 303.0 g versus 262.17 g (Khammarnia et al. 2024). In addition, the percentage contributions of carbohydrates, proteins, and fat to total energy intake during the third trimester of pregnancy and postpartum were within the acceptable ranges according to the DRIs (Institute of Medicine 2005).

Reducing carbohydrate and fibre intake from the third trimester of pregnancy to 8.5 months postpartum should raise concerns about a potential decrease in dietary quality (Oken et al. 2007). The type of carbohydrates consumed plays a significant role in maternal weight gain and foetal overgrowth, while low‐glycemic carbohydrates contribute to healthier maternal weight and normal infant weight. A diet rich in low‐glycemic index foods, such as fruits, vegetables, legumes, nuts, and whole grains, offers additional benefits, including the fibre's resistance to digestion by gastrointestinal enzymes (Oken et al. 2007; Afshin et al. 2017 Diet Collaborators 2019). In this study, participant's fibre intake (26.5 g in pregnancy and 22.1 g at the end of the postpartum period) was slightly below the AI (DRI) for pregnant women (28 g) and considerably below the AI for lactating women (29 g), indicating that women should increase their consumption of fibre‐rich foods (Institute of Medicine 2005). Adequate fibre intake during pregnancy has been linked to improved breastfeeding performance and infant neurodevelopmental outcomes (Miyake et al. 2023).

Total fat intake increased, including monounsaturated, polyunsaturated, and saturated fats, from pregnancy to 8.5 months postpartum. While mono‐ and polyunsaturated fatty acids are important during pregnancy to support maternal health and foetal brain and retina development (Oken et al. 2007), the rise in saturated fat intake warrants attention. Excessive intake of saturated fat is associated with an increased risk of cardiovascular disease, obesity, metabolic syndrome, insulin resistance, gestational hyperglycaemia, elevated blood pressure, and thrombosis (Oken et al. 2007; Astrup et al. 2021). Common dietary sources of saturated fat include dairy products, meat, coconut oil, and palm oil, and intake should not exceed 10% of the total energy. In this study, saturated fats contributed 10.6% of total energy intake during pregnancy and increased to 11.3% at 8.5 months postpartum. International and national guidelines recommend removing trans fats, reducing saturated fat consumption, and ensuring adequate intake of unsaturated fats (both mono‐ and polyunsaturated) during pregnancy (Brasil 2014; WHO 2016; Brasil 2021; WHO/FAO 2024). The trans‐fat intake in this cohort did not exceed 1% of the total energy during pregnancy and postpartum.

An alarming finding in this study was the low iron intake during pregnancy, with women reaching only 60.1% of the EAR. Iron is essential in haemoglobin production and oxygen transport, and its deficiency can have serious consequences. Globally, over 37% of pregnant women (approximately 32 million) are anaemic, with at least half of these cases due to iron deficiency (WHO 2024). Maternal anaemia is associated with increased risk of perinatal maternal and infant mortality and preterm delivery (Allen 2000; WHO 2016). Meeting micronutrient requirements through diet alone during pregnancy can be challenging, making supplementation often necessary. The WHO recommends daily iron and folic acid supplementation to prevent iron deficiency and the risk of neural tube defects during pregnancy. In Brazil, the National Iron Supplementation Programme (PNSF) recommends daily supplementation of 40 mg of elemental iron throughout pregnancy and 400 mcg of folic acid, starting at least 30 days before the planned pregnancy and continuing until the 12th week of gestation (Brasil 2022). In this study, 67.3% of women reported using supplemental iron, and 7.6% reported using supplemental folic acid during pregnancy. However, data on the dosage and frequency of iron supplementation were unavailable, though most participants likely adhered to the Ministry of Health's guidelines for iron and folic acid throughout pregnancy, as all participants had haemoglobin concentrations in the pulp space > = 10 g/dL. Regarding folate (vitamin B9) intake, there was a decrease from pregnancy to 8.5 months postpartum; however, the EAR adequacy remained above 80% during pregnancy and the postpartum period.

B‐complex vitamins are coenzymes in several intermediary metabolic pathways for energy production and blood cell formation. Therefore, a deficiency in these vitamins can impact cellular growth and nerve tissue development due to their high energy demand (Gernand et al. 2016). In this study, the pyridoxine intake (vitamin B6) did not change during follow‐up; however, it had an unsatisfactory adequacy, meeting only 53.1% of the EAR during pregnancy and dropping further to 47.5% of the EAR at the end of the 8.5 months postpartum. In contrast, vitamin B12 intake increased over time, with EAR adequacy exceeding 100% during the pregnancy and lactation periods, compared to other reports that have identified an insufficiency affecting 25% of pregnancies worldwide (Gernand et al. 2016; Sukumar et al. 2016).

This study revealed that vitamin D had the lowest adequacy levels during pregnancy, with < 30% of the EAR met, and remained low throughout the postpartum period. This outcome was already expected, considering that few food items naturally contain vitamin D in the Brazilian diet, such as salmon, tuna, and egg yolk (IOM 2011 Samimi et al. 2016; Figueiredo et al. 2018; Savard et al. 2019). In tropical countries like Brazil, sunlight exposure is the main source of vitamin D, yet dietary intake remains important. There is scarce data in the literature, but the ECLIPSES Study found a lower adequacy percentage (11.7% ± 6.9%) in pregnant women compared to the present study (Aparicio et al. 2020). It is essential to emphasise the importance of vitamin D for maternal and infant health, particularly during pregnancy, when the body's demand for this nutrient increases (Figueiredo et al. 2018; Arshad et al. 2022).

Another significant finding in this study was the sudden fall in the adequacy of vitamins A and E from pregnancy (87.6% and 61.3%, respectively) to postpartum M3 (46.0% and 45.0%, respectively). Vitamin A is essential for cell differentiation, the visual cycle, growth, reproduction, antioxidants, and the immune system. Additionally, foetal vitamin A reserves begin to form during the third trimester of pregnancy (WHO World Health Organization 1996). Vitamin E deficiency is also a burden, as it is associated with increased risk of pre‐eclampsia, premature rupture of amniotic membranes, and preterm delivery (Institute of Medicine 2000). The observed reduction in the intake of these vitamins may indicate a decrease in the consumption of meats, fish, and fish oil. Another longitudinal study on 793 pregnant women reported similar adequacy percentage (RDA) during pregnancy for vitamin A (79.8% ± 23.8%) and vitamin E (66.3% ± 7.6%), but higher levels during postpartum (83.5% ± 34.3% and 51.7% ± 5.6%, respectively) (Aparicio et al. 2020; Khammarnia et al. 2024).

The calcium intake decreased throughout the follow‐up, and its adequacy concerning the EAR was below 80%. This result aligns with other research (64.1% ± 27.1%) (Aparicio et al. 2020). Calcium is an essential nutrient for bone mineralisation and a critical intracellular component for maintaining cell membranes. It involves several biological processes, including muscle contraction, enzyme and hormone homoeostasis, neurotransmitter release, and nerve cell function (Samimi et al. 2016). The best sources of calcium are milk and dairy products, which can also be obtained from leafy green vegetables (Samimi et al. 2016; Tabela Brasileira de Composição de Alimentos TBCA 2023). The declining calcium intake and the rise in sodium intake postpartum reflect changes in dietary habits from pregnancy to lactation. Possible explanations relate to the search for practical foods, such as fast foods, which are convenient during the postpartum period when the woman is dedicated to caring for the baby and has little time to prepare meals. Excessive sodium or salt consumption is associated with several health problems, including hypertension, cardiovascular disease, kidney problems, and even cancer. The Food Guide for the Brazilian Population (2014) recommends that sodium intake be < 2 grams per day, equivalent to 5 grams of salt (Brasil 2014).

This study observed that pre‐pregnancy BMI, maternal age, education, family income, and marital status were factors associated with trajectories of macro‐ and micronutrient intake over time. A higher pre‐pregnancy BMI was associated with reductions in carbohydrate and fibre intake over time, as well as an increase in total, monounsaturated, and saturated fat intake. There is a lack of data in the literature to support these findings; however, it is noted that motherhood places increasing demands on time, mental and physical health, and financial resources, all of which may have a significant impact on eating behaviours postpartum (Saurel‐Cubizolles et al. 2000).

A meta‐analysis reported significant decreases in fruit and vegetable consumption and diet quality from pregnancy to postpartum, where fat contributions increase, and also found that women with lower education and lower income tended to have poor dietary behaviours postpartum (Lee et al. 2020). This finding aligns with the results of the present study, which showed that women with low educational attainment increased their sodium intake throughout the third trimester of pregnancy and up to 8.5 months postpartum. Additionally, women who lived without a partner experienced an increase in sodium and total fat intake. This could suggest that a weakened support network impacts women's food choices, as well as their limited access to healthier foods and information.

In this study, older and more educated women increased their calcium intake. Furthermore, mothers with greater age and income had an increase in vitamin C intake. It is well established that knowledge of dietary guidelines plays a crucial role in maternal health, with individuals from higher education groups and older age groups potentially being more aware of and adhering to nutritional recommendations (Jardí et al. 2019).

The strengths of this study are noteworthy: it has a prospective design with longitudinal analyses to verify the changes in nutrient intake over time; furthermore, nutrient intake was assessed through two R24h applied in the third trimester of pregnancy and at three moments postpartum, utilising a tablet app with photographic portion guides to facilitate interviewer recall and quantifying portions. All nutrients refer to the recommended daily intake. However, the study also has some limitations. First, the generalisability of the findings should be restricted to mother‐infant dyads, because women with comorbidities (e.g., gestational diabetes and pre‐eclampsia) were excluded during recruitment. This profile is coherent with the MILQ study objective, that is, establish reference values for micronutrients in human milk. However, adequate external validity is ensured, as the participants' profile is similar to that of those seeking primary care in the Unified Health System (SUS), which serves 75% of the Brazilian population (IBGE 2019). Losses to follow‐up also deserve attention, since they resulted in a reduced number of R24h throughout the study, especially from participants who responded to two instruments. Furthermore, some women responded by telephone and ultimately did not consult the album with portion sizes, which may lead to some error in estimating the amount consumed. Additionally, the calculation of sodium and potassium adequacy should be interpreted with caution, since no RDA or AI values are provided.

The trajectories of energy, macronutrient, and micronutrient intake from the third trimester of pregnancy to 8.5 months postpartum showed stability in energy and protein, a decline in carbohydrates and fibre, and discrete increases in total fat, monounsaturated, polyunsaturated, and saturated fat. In addition, there was a decline in the intake of most micronutrients. Sodium was the mineral with the most considerable increase over time. These results reinforce the need to improve nutritional management during pregnancy and postpartum, as they support the definition of strategies based on priorities. Specifically, they highlight the importance of targeted nutritional interventions, emphasising iron supplementation and promoting a diverse and healthy food consumption rich in fruits, vegetables, and animal products. In addition, educational and awareness‐raising strategies that involve women and their families, capable of reaching various education and income levels, from prenatal care on the role of food and nutrition during pregnancy and postapartum such to cooking workshops, preparatory courses for welcoming pregnant/puerperal women, are essential to promote adequate nutrition and prevent nutrient deficiencies and/or excess that adversely affect maternal and infant health.

## Author Contributions

The authors' responsibilities were as follows—Aline Yukari Kurihayashi, Gilberto Kac designed research. Aline Yukari Kurihayashi was primarily responsible for preprocessing the data, conducting trajectory optimisation and interpreting the results, with advisory inputs from Gilberto Kac, Daniela Polessa Paula and Bruna Celestino Schneider. Amanda Caroline Cunha Figueiredo, Gabriela Torres Silva, Adriana Divina de Souza Campos and Daniela de Barros Mucci provided data and tools. Aline Yukari Kurihayashi, Bruna Celestino Schneider, Amanda Caroline Cunha Figueiredo, Gabriela Torres Silva and Adriana Divina de Souza Campos drafted the manuscript. Bruna Celestino Schneider wrote the manuscript with critical comments from Gilberto Kac. Lindsay H. Allen and Gilberto Kac funding acquisition, conceptualisation, project administration, review and editing, and all authors: reviewed and approved the final manuscript.

## Conflicts of Interest

The authors declare no conflicts of interest.

## Supporting information


**Supplementary Figure 1:** Dataflow of the MILQ Study Brazil and maternal dietary records from pregnancy to nine months postpartum. MILQ Study Brazil, 2024. **Supplementary Table 1:** Characteristics of participants with dietary information on MILQ Study Brazil. MILQ Study Brazil, 2024. **Supplementary Table 2:** Associated factors with trajectories of energy, macro and micronutrients intake from pregnancy to nine postpartum. MILQ Study Brazil, 2024.

## Data Availability

The authors have nothing to report.
