# Supplementary material for: Trajectories of Dietary Energy, Macro and Micronutrient Intake From the Third Trimester of Pregnancy to 8.5 Months Postpartum Among Brazilian Women: The Mothers, Infants and Lactation Quality Study
Source: Matern Child Nutr. 2025 Aug 29;22(1):e70089. doi: 10.1111/mcn.70089 (PMC12893507; doi:10.1111/mcn.70089)
Supplement: Supplementary file 1 — Supplementary Figure 1: Dataflow of the MILQ Study Brazil and maternal dietary records from pregnancy to nine months postpartum. MILQ Study Brazil, 2024. Supplementary Table 1: Characteristics of participants with dietary information on MILQ Study Brazil. MILQ Study Brazil, 2024. Supplementary Table 2: Associated factors with trajectories of energy, macro and micronutrients intake from pregnancy to nine postpartum. MILQ Study Brazil, 2024. [file MCN-22-e70089-s001.docx]

24-hour recall

1 (n=145)

2 (n=107)

No answer to 24-hour recall

n=30 (17.1%)

24-hour recall

1 (n=108)

2 (n=83)

No answer to 24-hour recall

n=23 (17.6%)

24-hour recall

1 (n=196)

2 (n=150)

Excluded at C (n=34)^#^

- Eclampsia (n=8)

- Gestational diabetes (n=2)

- Pre-term/late-term (n=10)

- Low birth weight (n=11)

- High birth weight (n= 6)

- Congenital malformations (n=2)

Withdrawn (n= 32)

Excluded at M1(n=106)^##^

- Stopped or non-exclusive breastfeeding (n=90)

- Infant anthro (n=11)

- Supplements (n=6)

- Alcohol intake (n=5)

- Serious infant illness (n=2)

Withdrawn (n= 87)

Excluded at M2 (n=12)*

- Stopped breastfeeding (n=12)

- Infant anthro (n=4)

- Supplements (n=2)

- Alcohol intake (n=6)

Withdrawn (n= 39)

Excluded at M3 (n=11)**

- Stopped breastfeeding (n= 4)

- Infant anthro (n=2)

- Supplements (n=1)

- Alcohol intake (n=5)

Withdrawn (n= 30)

No answer to 24-hour recall

n=39 (16.6%)

No answer to 24-hour recall

n=125 (25.3%)

24-hour recall

1 (n=369)

2 (n=289)

Diet response rate (AN): 74.7% Diet response rate (M1): 83.4% Diet response rate (M2): 82.9% Diet response rate (M3): 82.4%

Diet follow up rate (AN): 100% Diet follow up rate (M1): 53.1% Diet follow up rate (M2): 39.3% Diet follow up rate (M3): 29.2%

28-35 gestational weeks

**(**baseline) < 1 1 - 3.4 3.5 - 5.9 6 - 8.5

Time (Months)

Supplementary Figure 1. Dataflow of the MILQ Study Brazil and maternal dietary records from pregnancy to nine months postpartum. MILQ Study Brazil, 2024.

^#^ 5 participants had two reasons or more.

^##^ 8 participants had two reasons or more.

* 12 participants had two reasons or more.

** 1 participant had two reasons or more.

Supplementary Table 1. Characteristics of participants with dietary information on MILQ Study Brazil. MILQ Study Brazil, 2024.

| Characteristics | **Baseline^*^**  **With answer to 24-hour recall**  (3^rd^ trimester pregnancy)  (n=369) | |  | **No answer to 24-hour recall at baseline ^**^**  (n=125) | | p value^***^ |
| --- | --- | --- | --- | --- | --- | --- |
|  | **n** | **(%)** |  | **n** | **(%)** |  |
| **Maternal age** (years) |  |  |  |  |  | <0.001 |
| 18-20 | 32 | 8.7 |  | 20 | 16.0 |  |
| 21-30 | 222 | 60.3 |  | 88 | 70.4 |  |
| 31-40 | 114 | 31.0 |  | 17 | 13.6 |  |
| **Marital status** |  |  |  |  |  | 0.760 |
| With partner | 300 | 81.3 |  | 88 | 80.0 |  |
| Without partner | 69 | 18.7 |  | 22 | 20.0 |  |
| **Skin color** |  |  |  |  |  | 0.194 |
| White | 68 | 18.4 |  | 21 | 19.1 |  |
| Black | 95 | 25.8 |  | 39 | 35.5 |  |
| Brown | 195 | 52.9 |  | 48 | 43.6 |  |
| Others (yellow/indigenous) | 11 | 2.9 |  | 2 | 1.8 |  |
| **Maternal education** (completed years) |  |  |  |  |  | 0.308 |
| <8 | 68 | 18.5 |  | 14 | 12.7 |  |
| 8-12 | 255 | 69.3 |  | 84 | 76.4 |  |
| >12 | 45 | 12.2 |  | 12 | 10.9 |  |
| **Parity** |  |  |  |  |  | 0.713 |
| Primiparous | 165 | 44.7 |  | 47 | 42.7 |  |
| Multiparous^#^ | 204 | 55.3 |  | 63 | 58.3 |  |
| **Pre-pregnancy BMI** (kg/m^2^) |  |  |  |  |  | 0.357 |
| Normal weight (18.5-24.9) | 225 | 61.0 |  | 82 | 65.6 |  |
| Overweight (25.0-29.9) | 144 | 39.0 |  | 43 | 34.4 |  |

^*^ Number of participants who responded to at least one 24-hour recall in the MILQ Study Brazil baseline.

^**^ Number of participants who were recruited for the MILQ but did not respond to the R24h or without valid dietary information.

^***^ Pearson's chi-square test between MILQ Study Brazil participants with and without answer to 24-hour recall.

^#^ Women with two children or more.

Supplementary Table 2. Associated factors with trajectories of energy, macro and micronutrients intake from pregnancy to nine postpartum. MILQ Study Brazil, 2024.

| Nutrients | β (z score) |  | p value ^†^ | 95% Confidence Interval | |
| --- | --- | --- | --- | --- | --- |
| Macronutrients |  |  |  |  |  |
| Carbohydrate |  |  |  |  |  |
| Pre-pregnancy BMI (kg/m^2^) | -0.02 |  | 0.007 | -0.03 | 0.00 |
| Total fat |  |  |  |  |  |
| Marital status |  |  |  |  |  |
| with partner | Ref. | | | | |
| without partner | 0.10 |  | 0.048 | 0.00 | 0.19 |
| Pre-pregnancy BMI (kg/m^2^) | 0.02 |  | 0.022 | 0.00 | 0.03 |
| Monounsaturated fat |  |  |  |  |  |
| Pre-pregnancy BMI (kg/m^2^) | 0.02 |  | 0.018 | 0.00 | 0.03 |
| Satured fat |  |  |  |  |  |
| Pre-pregnancy BMI (kg/m^2^) | 0.02 |  | 0.006 | 0.01 | 0.03 |
| Fiber |  |  |  |  |  |
| Pre-pregnancy BMI (kg/m^2^) | -0.03 |  | 0.049 | -0.05 | 0.00 |
| Micronutrients |  |  |  |  |  |
| Vitamin B2 |  |  |  |  |  |
| Age (y) | 0.02 |  | 0.011 | 0.00 | 0.03 |
| Vitamin E |  |  |  |  |  |
| Family income (tertile) |  |  |  |  |  |
| 1 | Ref. | | | | |
| 2 | 0.03 |  | 0.589 | -0.08 | 0.15 |
| 3 (higher) | 0.23 |  | 0.013 | 0.04 | 0.30 |
| Vitamin C |  |  |  |  |  |
| Family income (tertile) |  |  |  |  |  |
| 1 | Ref. | | | | |
| 2 | 0.22 |  | 0.023 | 0.02 | 0.26 |
| 3 (higher) | 0.36 |  | 0.001 | 0.09 | 0.37 |
| Age (y) | 0.02 |  | 0.031 | 0.00 | 0.02 |
| Calcium |  |  |  |  |  |
| Schooling (y) | 0.03 |  | 0.037 | 0.00 | 0.05 |
| Age (y) | 0.01 |  | 0.024 | 0.00 | 0.03 |
| Selenium |  |  |  |  |  |
| Pre-pregnancy BMI (kg/m^2^) | -0.02 |  | 0.025 | -0.04 | 0.00 |
| Age (y) | 0.02 |  | 0.005 | 0.00 | 0.03 |
| Sodium |  |  |  |  |  |
| Schooling (y) | -0.03 |  | 0.006 | -0.04 | -0.01 |
| Marital status |  |  |  |  |  |
| with partner | Ref. | | | | |
| without partner | 0.16 |  | 0.022 | 0.02 | 0.25 |

^†^ Generalized mixed-effect models likelihood p value.

Notes: Only those nutrients with a statistically significant association (p <0.005) are presented in this Table. All nutrients with a statistically significant (p <0.005) change over time were tested. The models were adjusted by usual energy (standardized), maternal age (years), maternal education (years), income (tertile), marital status (with/without partner), parity (primiparous/multiparous), and pre-pregnancy BMI (kg/m2).

Ref. : reference group
